# Quantitative analysis of human herpesvirus-6 genome in blood and bone marrow samples from Tunisian patients with acute leukemia: a follow-up study

**DOI:** 10.1186/1750-9378-7-31

**Published:** 2012-11-12

**Authors:** Nefzi Faten, Gautheret-Dejean Agnès, Ben Fredj Nadia, Abid Ben Salem Nabil, Zaier Monia, Khelif Abderrahim, Agut Henri, Feki Salma, Aouni Mahjoub

**Affiliations:** 1 Laboratory of Transmissible Diseases and Biological Active Substances, LR99-ES27, Faculty of Pharmacy, University of Monastir, Monastir, Tunisia; 2 UPMC Univ Paris 06, ER1 DETIV, Paris, France; 3 Laboratory of Virology, Pitié-Salpêtrière Hospital AP-HP, Paris, France; 4 Department of Clinical Hematology, Farhat Hached Hospital, Sousse, Tunisia; 5 Department of Clinical Biology, Faculty of Pharmacy, Monastir, Tunisia

**Keywords:** Human herpesvirus-6, Acute leukemia, Viral load, Bone marrow, Whole blood, Chemotherapy

## Abstract

**Background:**

Infectious etiology in lymphoproliferative diseases has always been suspected. The pathogenic roles of human herpesvirus-6 (HHV-6) in acute leukemia have been of great interest. Discordant results to establish a link between HHV-6 activation and the genesis of acute leukemia have been observed. The objective of this study was to evaluate a possible association between HHV-6 infection and acute leukemia in children and adults, with a longitudinal follow-up at diagnosis, aplasia, remission and relapse.

**Methods:**

HHV-6 load was quantified by a quantitative real-time PCR in the blood and bone marrow samples from 37 children and 36 adults with acute leukemia: 33 B acute lymphoblastic leukemia (B-ALL), 6 T acute lymphoblastic leukemia (T-ALL), 34 acute myeloid leukemia (AML).

**Results:**

HHV-6 was detected in 15%, 8%, 30% and 28% of the blood samples at diagnosis, aplasia, remission and relapse, respectively. The median viral loads were 138, 244, 112 and 78 copies/million cells at diagnosis, aplasia, remission and relapse, respectively. In the bone marrow samples, HHV-6 was detected in 5%, 20% and 23% of the samples at diagnosis, remission and relapse, respectively. The median viral loads were 34, 109 and 32 copies/million cells at diagnosis, remission and relapse, respectively. According to the type of leukemia at diagnosis, HHV-6 was detected in 19% of the blood samples and in 7% of the bone marrow samples (with median viral loads at 206 and 79 copies/million cells, respectively) from patients with B-ALL. For patients with AML, HHV-6 was present in 8% of the blood samples and in 4% of the bone marrow samples (with median viral loads at 68 and 12 copies/million cells, respectively). HHV-6 was more prevalent in the blood samples from children than from adults (25% and 9%, respectively) and for the bone marrow (11% and 0%, respectively). All typable HHV-6 were HHV-6B species. No link was shown between neither the clinical symptoms nor the abnormal karyotype and HHV-6 activation. A case of HHV-6 chromosomal integration was shown in one patient with AML.

**Conclusion:**

This study confirms the absence of role of HHV-6 in the genesis of acute leukemia but the virus was reactivated after chemotherapy treatment.

## Background

Human herpesvirus-6 (HHV-6) belongs to the *Roseolovirus* genus of the *Betaherpesvirinae* subfamily of the *Herpesviridae* family. HHV-6 was first isolated from B-lymphocytes of patients with lymphoproliferative disorders [1]. HHV-6 is separated into two major subgroups, novo designed species, HHV-6A and-6B on the basis of distinct genetic, immunological and biological characteristics [2].

HHV-6 genome is a linear, double-stranded DNA molecule, 160 to 162 kpb in size, flanked by terminal direct repeats (DRL and DRR) of 8 to 9 kpb. The unique long (UL) region is interrupted by three intermediate repeats, R1, R2 and R3, in the immediate-early A region. The genes in UL are termed U1 to U100 and open reading frames (ORFs) within the direct repeats are designated as DR1 to DR7 [3].

The cellular receptor of HHV-6 is CD46, expressed on the surface of normal and malignant cells as well as on leukemic cells [4]. HHV-6 oncogenic potential was demonstrated *in vitro* in NIH3T3 cells [5], and ORF-1, also referred to as DR7, was identified as an oncogene. The binding of DR7 to the tumor suppressor protein p53 and the inhibition of p53 activated transcription were evidenced. Along with the detection of DR7 in malignant tissues, these activities may indicate a role of DR7 in human cancer [6]. Recently, the presence of HHV-6 and the expression of the viral DR7B oncoprotein in Reed-Sternberg cells from Hodgkin’s lymphoma patients have been reported [7].

An interesting feature is the integration of HHV-6 (CI-HHV-6) DNA into the cellular genome which is found in about 1% of the general population. This phenomenon was described in patient with acute lymphoblastic leukemia and is transmitted from parents to children through generations [8]. Interestingly, a careful review of the literature shows some pathologies like hematological neoplasia, appear overrepresented [9]. The insertion of the whole genome of HHV-6 (162 kpb) within the telomeric regions could have consequences in terms of cell physiology.

Acute leukemia is a multifactorial disease where an infectious etiology is suspected. The role of HHV-6 in the development of hematological disease is of continuous interest. Discordant results to establish a link between HHV-6 infection and the genesis of acute leukemia were observed in a prospective study. Human herpesvirus-6 was found in leukemic cells of patients with T-ALL [10]. The median viral loads were at 1,512 copies/million cells for patients with lymphoproliferative disorders [11] and at 1,374 copies/million cells for patients with B-cell malignancies [12]. On the contrary, HHV-6 was similarly detected in patients with acute lymphoblastic leukemia and controls [13], and the virus was more present in children with acute lymphoblastic leukemia at complete remission than at diagnosis [14].

The present work is aimed at carrying out a follow-up study and evaluating a possible association of HHV-6 in children and adults with acute leukemia at diagnosis, aplasia, remission and relapse.

## Results

### Analysis of HHV-6 detection and quantitation

Overall, HHV-6 was more prevalent in blood (70% of positive samples) than in bone marrow (30% of positive samples) (*p* = 0.03) with a higher median viral load (136 and 12 copies/million cells, respectively, *p* = 0.02).

The detection of HHV-6 showed a significant difference among the different clinical stages (*p* = 0.02, Table 1).

**Table 1:** Detection and quantitation of HHV-6 DNA in blood and bone marrow samples

**Sample type**	**Detection of HHV-6 (%) and quantitation (range) expressed as copies/million cells at**							
	**Diagnosis**		**Aplasia**		**Remission**		**Relapse**	
	**Detection**	**Quantitation**	**Detection**	**Quantitation**	**Detection**	**Quantitation**	**Detection**	**Quantitation**
**Blood**	9 [15]^a,b^	138 (12 – 6 074074)	5 [8]^c,d^	244 (68 – 6 546567)	18 (30) ^a,c^	112 (26 – 6 675980)	11 (28) ^b,d^	78 (19 – 8 533719)
**Bone marrow**	3 [5]^a,b^	34 (12 – 123)	Nc	Nc	9 [16]^a^	109 (10 – 245)	8 [17]^b^	14 (4 – 78)
**All types of samples**	12 [10]	206 (12 – 6 074074)	5 [8]	244 (68 – 6546 567)	27 (26)	114 (10 – 6 675 980)	19 (26)	14 (4 – 8 533 719)

Nc: Not collected; ^a^*p* = 0.04 for blood; *p* = 0.01 for bone marrow; ^b^*p* = 0.06 for blood; *p* = 0.03 for bone marrow; ^c^*p* = 0.005; ^d^*p* = 0.02.

In the blood samples, HHV-6 was detected in 15%, 8%, 30% and 28% at diagnosis, aplasia, remission and relapse, respectively. HHV-6 detection was significantly higher at remission than at diagnosis (*p* = 0.04) or during aplasia (*p* = 0.005), and higher at relapse than at diagnosis (*p* = 0.06) or during aplasia (*p* = 0.02).

In the bone marrow samples, HHV-6 was detected in a similar way as in the blood samples. It was not possible to collect bone marrow samples from patients at the time of aplasia. HHV-6 detection rate was different during the remaining three stages (*p* = 0.04). As observed for the blood samples, HHV-6 detection rate increased in samples at remission (*p* = 0.01) and at relapse (*p* = 0.03) compared to diagnosis.

Statistically, no significant viral load differences were observed between samples at different disease stages (Table 1).

When considering the age groups some significant differences were evidenced. Thus, the overall prevalence of HHV-6 detection in the blood samples from children (25%) was higher than that observed in adults (8%) (*p* = 0.005), with median viral loads of 194 and 68 copies/million cells, respectively. In the bone marrow samples, HHV-6 was more prevalent in children (17%) than in adults (3%) (*p* = 0.001), with median viral loads at 12 and 68 copies/million cells, respectively. The present results also differed from children to adults according to the stage of disease. Thus, for the blood samples from children, HHV-6 was detected in 25%, 13%, 35% and 24% at diagnosis, aplasia, remission and relapse, respectively (with viral loads at 274, 136, 118 and 125 copies/million cells, respectively). As for the blood samples from adults, HHV-6 detection rates were lower (9%, 3%, 18% and 19% at diagnosis, aplasia, remission and relapse, respectively), with also lower viral loads (68, 68, 88 and 50 copies/ million cells, respectively). The same tendency was observed in the bone marrow samples. For children, HHV-6 was detected in 11%, 17% and 34% at diagnosis, remission and relapse, respectively (with viral loads at 34, 125 and 30 copies/million cells, respectively). For adults, HHV-6 detection rates were 0%, 9% and 8% at diagnosis, remission and relapse, respectively, with also lower viral loads at remission and relapse (94 and 14 copies/ million cells, respectively).

The results according to the type of leukemia at diagnosis are presented in Table 2. When considering the frequency of HHV-6 detection according to the different periods, HHV-6 was more present at remission and relapse than at diagnosis and aplasia whatever the type of leukemia considered. For patients with B-ALL, HHV-6 was detected in 12%, 27% and 23% of the blood samples (median viral loads at 388, 97 and 92 copies/ million cells, respectively) at aplasia, remission and relapse, respectively. In the bone marrow samples, similar detection rates were observed: 24% and 28% (median viral loads at 109 and 31 copies/million cells) at remission and relapse, respectively.

**Table 2:** Detection and quantitation of HHV-6 according the type of acute leukemia at diagnosis

**Pathology**	**Number of samples (blood/bone marrow)**	**Detection of HHV-6 in blood/bone marrow (%)**	**HHV-6 median load expressed as copies/million cells (range) in blood**	**HHV-6 median load expressed as copies/million cells (range) in bone marrow**
**B-ALL**	62 (31/30)	6/2 (19/7)	206 (41 – 1 225)	79 (34 – 123)
**T-ALL**	7 (5/2)	0	0	0
**AML**	64 (37/27)	3/1 (8/4)	68 (12 – 6 074 074)	12
**All patients**	133 (73/59)	9/3 (15/5)	206 (12 – 6 074 074)	34 (12–123)

B-ALL: B Acute Lymphoblastic Leukemia; T-ALL: T Acute Lymphoblastic Leukemia; AML: Acute Myeloid Leukemia.

For patients with AML, HHV-6 was equally detected in 19% of the blood samples (median viral loads at 121 and 49 copies/million cells) at remission and relapse, respectively. In the bone marrow samples, similar detection rates were observed: 15% and 28% (median viral loads at 172 and 32 copies/million cells) at remission and relapse, respectively. During aplasia, HHV-6 was detected in 6% of the blood samples with a viral load at 156 copies/million cells.

### HHV-6 typing

In 57 samples, HHV-6 was typed as HHV-6B species. However, it was not possible to type the detected virus in six cases.

### Genetic analysis

Among 60 patients with a karyotype that was determined at diagnosis, 24 had a normal karyotype and 36 had an abnormal one. HHV-6 was detected in four patients with an abnormal karyotype (11%) and in five patients with a normal karyotype (15%), without any statistical correlation between HHV-6 detection and abnormal karyotypes (*p* = 0.4). The representative abnormal karyotypes of HHV-6 positive samples were t(8;5;17), t(8;16), t(9;22) (4;9), t(8;21), t(15;17), t(8;21) –Y, t(2;6;14), t(12;15;17), t(18;21), del(12p), del(11p), inv [18] + 8del (7q), del(9q) –Y, inv[18] + 5 + 21, del[11] + 21, hyperdiploidy.

### Correlation between HHV-6 detection and hematological clinical disorders

The relationship analysis did not show any correlation between hematological clinical disorders and HHV-6 detection rate (leucopenia, *p* = 0.8; lymphopenia, *p* = 0.7; agranulocytosis, *p* = 0.6; severe lymphopenia, *p* = 0.6; asthenia, *p* = 0.1; fever, *p* = 0.3).

### Chromosomal integration

A high HHV-6 DNA load in blood at diagnosis (6,074,074 copies/million cells), at aplasia (6,546,567 copies/million cells), at remission (6,675,980 copies/million cells) and at relapse (8,553,719 copies/million cells) was observed in one patient with AML. CI-HHV-6 was suspected and confirmed by measuring the viral load in hair follicles (7,583,774 copies/million cells). HHV-6 B species was identified.

This patient was a 50-year-old man with type 2 AML, without a family history of leukemia. At diagnosis, the patient had fever, asthenia, nausea, pancytopenia, anemia and leucopenia. The percentage of blasts was 44% in blood and 49% in the bone marrow and the karyotype was normal 46, XY. Later, he received chemotherapy and was in remission.

When the blood samples of the patient’s family members (wife, son and daughter) were analyzed to search for HHV-6 DNA chromosomal transmission, a high HHV-6 DNA load was detected for the son (3,456,376 copies/million cells), and a low and negative viral load for the daughter (18 copies/million cells) and the wife, respectively.

## Discussion

The hypothesis of a potential role of HHV-6 infections in acute leukemia in children and adults was verified here by carrying out a follow-up study on Tunisian patients using real-time PCR for virus quantitation in peripheral blood and bone marrow samples at diagnosis, aplasia, remission and relapse.

To our knowledge, this is the first follow-up study carried out on Tunisian children and adults comparing, for the same patients, HHV-6 detection rate and viral load in peripheral blood and bone marrow samples at different stages of acute leukemia.

In the present study, HHV-6 was detected in 17% of the blood samples from children and adults with ALL at diagnosis with a median viral load at 206 copies/million cells. The results obtained by Hermouet et al. evidenced a higher HHV-6 detection rate (36%) with also a higher viral load at 1,374 copies/million cells [12]. These differences may be due to variability in patient cohorts, and to different qPCR sensitivities.

In the bone marrow samples at diagnosis, the current results are in accordance with previous findings obtained by Seror et al. who detected HHV-6 in 17% of bone marrow samples with a viral load at 23 copies/million cells [14].

The current data showed the presence of HHV-6 in 5% of the bone marrow samples from adults and children. As observed in the blood samples, these results greatly differed from those obtained by Hermouet et al. who detected HHV-6 in 41% of bone marrow samples with a viral load at 206 copies/million cells [12].

At remission, HHV-6 was significantly more prevalent in blood and bone marrow than at diagnosis, confirming the high frequency detection of HHV-6 in patients at remission [14].

The present results evidenced the low detection of HHV-6 in the bone marrow samples at diagnosis where the median frequency of blastic cells was 81% (22% - 99%), but the presence of the virus at remission. This proves the absence of HHV-6 in leukemic cells. The virus detected was probably present in normal lymphocytes that were found at low percentages at diagnosis of leukemia. This can also explain the low viral load observed.

Andre-Garnier et al. demonstrated that HHV-6 can infect differentiated cells (lymphocytes) and very immature cells (early bone marrow progenitors CD34+) and not lymphoblasts [15]. The current study demonstrated the presence of HHV-6 in bone marrow at remission at a higher rate than at diagnosis. This result is in favor of the presence of the virus in CD34+ cells or in circulating cells carrying HHV-6 that go back into the bone marrow.

The higher prevalence of HHV-6 infection in case of B-ALL than AML or T-ALL and in children compared to adults may be due to primary HHV-6 infection frequent at childhood and in the higher occurrence of B-ALL that constitutes the most common cancer in childhood. It would be interesting to analyze surface markers of B leukemic cells sorted and search for the presence of HHV-6 genome.

HHV-6 was detected in samples from patients at remission and relapse. Reactivation of latent HHV-6 during the course of anti-leukemia treatment may occur and can be explained by the suppression of the immune functions in aggressively-treated patients. However, HHV-6 was detected only in 8% of patients at aplasia when immunosuppression is at its highest. This can be explained by the low number of CD3+ cells or reactivation of the virus in other sites of the organism.

The identification of only HHV-6 B species in this study is in accordance with the results obtained by seror al. [14], but differs from those obtained by Hermouet et al. who identified 89% of HHV-6A species, and 11% of HHV-6B [12]. This may be linked to the high number of children included in our study, the frequent occurrence of HHV-6 infections during childhood and to inter-laboratory technical discrepancies.

The clinical symptoms related to HHV-6 were studied to establish a link with HHV-6 activation but no correlation was observed in these patients.

This study reported a case of CI-HHV-6 in an adult patient with AML. The 1.2% prevalence of CI-HHV-6 observed in our studied population is in agreement with other studies in which CI-HHV-6 was present in 1.5% of children with leukemia [16] and in 0.2 - 2.9% of different human populations [17-20]. HHV-6 genome was transmitted from father to son who does not have any signs of a hematological disease.

## Conclusions

In conclusion, this study supports a viral reactivation after chemotherapy treatment rather than a causal role of HHV-6 in the genesis of acute leukemia.

## Methods

### Ethical approval and consent

This study was approved by the ethics committee and medical research of the Farhat Hached University hospital, Sousse, Tunisia (Reference number: 12/2012). Written informed consent was obtained from each case patient for publication of this report.

### Patients and clinical samples

The present prospective study was carried out using samples from patients with acute leukemia consulting the Clinical Hematology Department (Farhat Hached Hospital, Sousse, Tunisia). The data concerning each patient were collected (Name, Sex, Age, and Address) (data not shown). The follow-up study was performed during the four principal stages of disease and treatment (diagnosis, aplasia, remission and relapse).

Initially, a total of 73 patients were included in the follow-up study. However, 13 out of them did not continue the experiment and were excluded from the statistical analysis. The remaining 60 patients continued the follow-up until the end of the experiment (Table 3). They consisted of 30 B acute lymphoblastic leukemia (B-ALL), 0 T acute lymphoblastic leukemia (T-ALL), and 30 acute myeloid leukemia (AML) at diagnosis. The blood samples were taken from all patients whereas the bone marrow sampling was not possible for all. Altogether, it was possible to collect bone marrow samples only from 59 out of 73 patients at diagnosis (Table 3).

**Table 3:** Distribution of patients according to periods, age group and leukemia types

**Sample Type**	**Periods**				
	**Diagnosis (Ch/Adlt)**	**Aplasia (Ch/Adlt)**	**Remission (Ch/Adlt)**	**Relapse (Ch/Adlt)**	**All periods (Ch/Adlt)**
**Blood**	73 (37/36)	60 (30/30)	60 ( 30/30)	39 (19/20)	232 (116/116)
**Bone marrow**	59 (29/30)	Nc	45 (24/21 )	35 (17/18)	139 (70/69)
**All type of samples**	132 (66/76)	60 (30/30)	105 (54/61)	74 (36/38)	371 (186/185)

Ch: Children; Adlt: Adult; Nc: Not collected.

Sixty samples were collected at aplasia, 30 samples from patients with B-ALL and 30 from patients with AML. At remission, 60 samples were collected from the same patients and at relapse, 23 samples were collected from patients with B-ALL and 16 from patients with AML.

At diagnosis, the median percentage of blastic cells was 54% (range 25 – 96%) in blood and 81% (range 22 – 99%) in bone marrow. Remission was established when leukemic cells were less than 5% in bone marrow samples. The patient was considered at relapse when the percentage of leukemic cells exceeded 5% in the bone marrow samples. Thirty-nine patients out of 60 were found at the stage of relapse (Table 3).

The median age of the patients was 21 years (range 1 – 66 years). The sex ratio (male/female) was 0.9.

### DNA extraction

Total DNA (viral and cellular) from blood and bone marrow samples was extracted from 200 μL of EDTA-treated whole blood or bone marrow using QIAamp DNA Blood Mini kit (Qiagen, Courtaboeuf, France) according to the manufacturer’s instructions. DNA was eluted in 200 μL of elution buffer and stored at −80°C until use.

### HHV-6 and cellular DNAs quantitation

The quantitation of the HHV-6 genome was carried out using Taq 1, Taq 2 primers and H6S probe (Table 4), as described previously [21]. In addition, the amplification of the human albumin gene was performed using Alb-forward, Alb-reverse primers and Alb-probe (Table 4), as described previously [22].

**Table 4:** Oligonucleotides used

**Primers and probes**	**Orientation**	**Sequence (5’ 3’)**	**References**
**HHV-6 detection**			
Taq1	+	GACAATCACATGCCTGGATAATG	[21]
Taq 2	-	TGTAAGCGTGTGGTAATGGACTAA	
H6S probe		FAM-AGCAGCTGGCGAAAAGTGCTGTGC-TAMRA	
**Human albumin gene**			
Alb-forward	+	GCTGTCATCTCTTGTGGGCTGT	[22]
Alb-reverse	-	ACTCATGGGAGCTGCTGGTTC	
Alb-probe		FAM-CCTGTCATGCCCACACAAATCTCTCC-TAMRA	
**HHV-6 (A and B)**			
HHV-6A	-	TGGTAATGGACTAATTGTGTGTTGTTTTA	[23]
HHV-6B	**-**	TGGTAATGGACTAAGTGTGCGTTATTTTC	

The reaction was carried out in a final volume of 50 μL containing 25 μL of Taq Man Universal PCR MasterMix (Applied Biosystems-Applera, Villebonsur-Yvette, France), 200 nM of each primer, 100 nM of specific probe, 10 μL of sterile water and 10 μL of DNA extract. The amplification step was carried out through a denaturation step at 50°C for 2 min and at 95°C for 10 min followed by 45 cycles (95°C for 15 sec, 60°C for 1 min). Ten-fold serial dilutions of HHV-6 DNA from HST cultured in MT4 cells or human DNA (Roche Diagnostics, Meylan, France) were used to establish standard curves. The detection limit was 10 copies of viral genomic equivalents per reaction.

The HHV-6 load was expressed as the number of viral genomic equivalent copies per million of cells (copies/million cells).

### HHV-6 typing

The identification of species such as HHV-6 A or B was performed for each positive sample. HHV-6 typing was carried out using HHV-6A and HHV-6B specific primers (Table 4), as described previously [23]. The detection limit of this real-time PCR method was 10 copies of viral genomic equivalents per reaction. In all experiments, the reactions were carried out using PE-Applied Biosystem Sequence Detector 7000 or 7500.

### Statistical analysis

The Statview software (release 5.0, SAS institute) was used to perform descriptive statistics, Mann–Whitney test, Fisher’s test and ANOVA, as appropriate. *p-*value < 0.05 was considered as statistically significant.

## Abbreviations

HHV-6: Human Herpesvirus-6; B-ALL: B acute lymphoblastic leukemia; T-ALL: T acute lymphoblastic leukemia; AML: Acute myeloid leukemia; UL: Unique long; DRR: Direct Repeat Right; DRL: Direct Repeat Left; ORF: Open reading frames:; CI-HHV-6: Chromosomal Integration of HHV-6; t: Translocation; inv: Inversion; del: Deletion.

## Competing interests

The authors have no competing interests.

## Authors’ contributions

NF carried out qPCR, analyzed, interpreted results and drafted the manuscript; GDA participated in study design, analyzed, interpreted results and helped revise the manuscript; BFN helped to DNA extraction; ABSN helped to manuscript writing; ZM participated to samples and clinical data collection; KA participated to samples and clinical data collection, HA analyzed and interpreted results; FS analyzed and interpreted results; OM participated in study design, analyzed and interpreted results. All authors have read and approved the final manuscript.
